# Physical and Physiological Indicators of Welfare in Guinea Pigs (*Cavia porcellus*) Serving as Ambassador Animals

**DOI:** 10.3390/ani10050815

**Published:** 2020-05-08

**Authors:** David M. Powell, Corinne P. Kozlowski, John Clark, Alice Seyfried, Eli Baskir, Ashley D. Franklin

**Affiliations:** 1 Department of Reproductive and Behavioral Sciences, Saint Louis Zoo, 1 Government Drive, Saint Louis, MO 63110, USA; kozlowski@stlzoo.org (C.P.K.); baskir@stlzoo.org (E.B.); 2 Children’s Zoo, Saint Louis Zoo, 1 Government Drive, Saint Louis, MO 63110, USA; clark@stlzoo.org (J.C.); seyfried@stlzoo.org (A.S.); 3 AZA Reproductive Management Center, Saint Louis Zoo, 1 Government Drive, Saint Louis, MO 63110, USA; franklin@stlzoo.org

**Keywords:** welfare, glucocorticoids, zoo, domestication, ambassador animal

## Abstract

**Simple Summary:**

Ambassador animals give zoo and aquarium visitors the opportunity to connect with nature, but it is important to assess the welfare of these animals while serving in this role. We conducted a study to determine whether guinea pigs housed in a publicly accessible habitat and serving as ambassador animals demonstrated any differences in welfare indicators compared to periods when they are housed off exhibit. We found that individual differences were the largest drivers of glucocorticoid levels, while sex and on- or off-exhibit housing did not have significant effects on mean levels. There were sex differences in variation of glucocorticoid levels. Moving between habitats did not elicit a significant hormone response except when females were moved off exhibit. Neither the amount of handling the animals received nor closure of the exhibit affected hormone levels. Guinea pig body weights were lower on average when on exhibit but did not otherwise significantly differ. Our results suggest that a monthly rotational schedule of exposure to the public does not negatively impact the physical and physiological indicators of welfare studied.

**Abstract:**

Special encounters that allow contact between animals and guests are common in zoos and aquariums. Visitors to the Saint Louis Zoo may touch guinea pigs serving as ambassador animals. We evaluated two welfare indicators in ambassador guinea pigs by comparing glucocorticoid levels and body weights between periods when guinea pigs lived in a habitat accessible to the public and while off exhibit. Mean glucocorticoid levels did not differ between sexes or between on- and off-exhibit periods. There was significant individual variation, and females demonstrated greater variation than males. While on exhibit, glucocorticoid levels slightly but significantly increased in males and decreased in females. Moving guinea pigs between habitats only elicited a significant glucocorticoid response when females were moved off exhibit. Temporary closures of the exhibit had no effect on glucocorticoid levels in either sex. Analyses of the impact of handling rates on males found no impact on glucocorticoid levels. Guinea pigs’ body weights were lower while on exhibit. We conclude that guinea pigs serving as ambassador animals at the Saint Louis Zoo demonstrate comparable physiological profiles while on and off exhibit and, when used in a rotational schedule, are a suitable species for animal encounters involving contact with the public.

## 1. Introduction

Research and policy interest in the welfare of ambassador animals is stimulated by two salient features of their lives: First, ambassador animals come into direct or close contact with the public on a regular basis, while either in their habitats (e.g., for domestic animals in “petting zoos”) or when brought into the public’s space. Many different species are used as ambassador animals, and one might predict that domesticated species would be more comfortable with close, controlled interactions with the public. However, it is not clear whether interactions with the public have positive, negative, or neutral outcomes for individuals serving as ambassadors and whether domestication affects these outcomes.

The second salient difference is that ambassador animals often live in spaces that are different from those occupied by animals on exhibit. These spaces may differ in sizes, naturalistic features, and proximity to other species. Ambassador animals may move among several enclosures regularly as part of educational programs, whereas exhibit animals typically occupy only one habitat. However, this difference in living areas is not always the case; some facilities house their ambassador animals in traditional zoo exhibits but remove them from these spaces temporarily to participate in programming. In some cases, ambassador animals live full-time in enclosures that are virtually identical to traditional exhibits and may encounter visitors either at the perimeters of such enclosures or within them when visitors are allowed to enter.

To date, relatively few studies of ambassador animals have been published, and the results appear to be species- or situation-specific. Baird et al. (2016) found that glucocorticoid levels increased in several species with increasing amounts of handling for ambassador or husbandry purposes [1]. Additionally, handling for any purpose was positively correlated with undesirable behaviors in armadillos (*Tolypeutes*, *Chaetophractus*, *Dasypus*, and *Euphractus* spp.). Miller, Mellen, Greer, and Kuczaj (2011) found that participation in shows or interactions with humans was associated with either no change or positive changes in behavior in bottlenose dolphins (*Tursiops truncatus*) [2], and Clegg, Rödel, Boivin and Delfour (2018) found that dolphins perceived human–animal interactions as rewarding, as evidenced by increased anticipatory behavior [3]. Majchrzak, Mastromonaco, Korver and Burness (2015) found that glucocorticoid levels did not differ when domestic camels (*Camelus dromedarius*) were in a pasture, standing in a ride area, or providing rides at either high or low frequency, and glucocorticoid levels during the ride season were lower than before or after this period [4]. Farrand, Hosey, and Buchanan-Smith (2014) found no behavioral changes suggestive of poor welfare in domestic ungulates in a petting zoo or evidence that grooming by the public negatively impacted the animals [5]. Anderson, Benne, Bloomsmith and Maple (2002) found that the availability of a retreat area reduced “undesirable” behavior in domestic ungulates in a petting area, but this effect depended on the design of the retreat area [6].

Glucocorticoids are often used as indicators of animal welfare because levels reflect the body’s physiological preparation for challenge [7]. It is important to understand that these hormones do not increase only in response to negative stress; they also increase in response to the anticipation of positive activities such as play [8], sexual behavior [9], and in response to environmental enrichment [10]. However, chronic elevation of glucocorticoids or failure of the hypo-thalamic-pituitary axis to respond to challenging (“stressful”) stimuli reflects compromise of an animal’s ability to cope with stimuli in its environment [11] and could result in compromised immune function or reproduction.

Here, we describe patterns of glucocorticoid production and body weight as potential welfare indicators in guinea pigs (*Cavia porcellus*) serving as ambassador animals at the Saint Louis Zoo. Guinea pigs do not exist in the wild and are domestic descendants of the wild cavy [12]. Wild cavies are found in dense ground vegetation and live in small harem groups of a single male and one or more females and their offspring [13]. Males are generally intolerant of one another and females exhibit a linear dominance hierarchy [12]. Guinea pigs were first domesticated as early as 5000 BC in the Andean region of South America by tribes using them as food and in cultural practices [14]. They were introduced to Europe in the sixteenth century and rapidly became a popular pet [12], though selective breeding that gave rise to some of the domestic breeds found today started in approximately 1200 AD [15], resulting in a behavioral phenotype that today is rather docile and tractable. The domestication process has also affected the social system of the species in that multi-male groups are behaviorally compatible when either the males are descendants of the main breeding male or when the animals have sufficient space to assort themselves into subgroups [12]. It is recommended to house guinea pigs in either male-female pairs, single male harems, large multi-male, multi-female groups, or all-female groups [12]. Pairs of intact males may be housed together, but aggression and intolerance are increased in larger groups. Guinea pigs form linear, within-sex dominance hierarchies with little to no overt aggressive behavior [12].

In this study, we sought to answer several questions related to welfare indicators in guinea pigs managed as ambassador animals. First, we wanted to determine if glucocorticoid production differs between the female and castrated male groups, which are managed separately at the Zoo. Bauer et al. (2008) found the fecal glucocorticoid levels were not significantly different between females and intact male guinea pigs [16], though earlier studies found that plasma glucocorticoid levels were higher in females compared to males [17]. In other species, similar levels of cortisol have been described for females and castrated males [18]. Second, we asked whether glucocorticoid levels of guinea pigs are higher when living on exhibit and participating in programs compared to when animals are off exhibit. We hypothesized that glucocorticoid levels might be higher when the animals are on exhibit because they would be exposed to zoo visitors while in the public habitat and may experience more physiological arousal while being handled for contact sessions with the public. This stimulus does not occur when off exhibit. Rodents may respond to transport with glucocorticoid responses [19], and animals may respond to changes in housing or environment with changes in glucocorticoids [20]. Responses to transport may be reduced however, when animals have previous exposure to the stimulus [21]. Thus, we also investigated whether the transitions of moving on from off exhibit or vice versa result in a change in glucocorticoid levels. Related to this, Walters et al. (2012) demonstrated that glucocorticoids were still increasing four days after a four-day transport in laboratory housed guinea pigs [22] and Stemkens-Sevens et al. (2009) found that guinea pigs required a 10 to 12 day period for heart rate, body temperature, and activity levels to return to pre-transport levels; the transports lasted between two and eight hours in that study [23]. We analyzed glucocorticoid levels over time during a period of housing, on or off exhibit, to determine whether guinea pigs show evidence of habituation in terms of glucocorticoid levels. A planned closure of the public exhibit was utilized to investigate whether glucocorticoid levels differed in the public exhibit when it was open versus closed. This allowed us to examine the impact of the presence of visitors [24], separate from having contact with the animals.

We considered body weight as a welfare indicator because the ability to maintain body weight reflects appropriate physiological function; significant increases or decreases in body weight could reflect dysfunction. Previous work in guinea pigs has found mixed results with regard to the impact of stressors on body weight [25,26,27] with some animals showing evidence of weight loss as a result of stressful social stimuli whereas other do not. Loss of body weight has been documented in guinea pigs during animal transport as well [23]. We used body weights collected periodically during the study to assess whether this welfare indicator was impacted by public contact.

## 2. Materials and Methods

Longitudinal fecal samples were collected from 16 guinea pigs housed in single-sex groups over a five-month period in 2017 that included two on-exhibit and two off-exhibit 30-day periods for each sex, plus an additional on-exhibit period for the males. Due to differences in handling rates for males in their first on-exhibit period (see below), this first period was not included in all analyses, as described below. This study was approved by the Saint Louis Zoo’s research review committee (19-01 and 19-05).

### 2.1. Study Animals and Housing

Our observational units were 8 female and 8 castrated male guinea pigs living in 2 separate single-sex groups. These sixteen animals were selected from a group of 21 females and 19 males housed at the Zoo. Both groups contained additional animals of the designated sex, but the subjects in this study were selected because they were easily identified by color pattern. Guinea pigs in the collection were obtained as donated pets, from private breeders, or were born at the zoo. Their individual histories prior to arrival at the zoo were unknown. The animals ranged in ages from 2 months to 2 years at the start of the study. Guinea pigs are sexually mature at one month (females) and two to three months (males) of age [12]. All had been at the zoo since birth or spring of 2015 and thus had been part of the public contract program for at least one year except for one two-month old male. When animals are obtained from outside sources, they undergo a gradual introduction process to groupmates. All animals new to the contact program are acclimated to the procedure via practice sessions of increasing duration off exhibit to habituate them to the zoo’s contact program. Animals that show repeated signs of fear, distress, or aggression when involved in a contact session are removed from the program, but this occurrence is exceedingly rare.

The guinea pig groups alternated approximately 30-day periods between two habitats. The rotation of animals between habitats was accomplished in less than an hour in the morning before the zoo opened. The on-exhibit habitat measured approximately 3.4 m by 2.3 m and was located indoors in a public area of the Saint Louis Zoo Children’s Zoo (CZ). Approximately 50% of the habitat perimeter was a low wall (35.5 cm high) with a wide flat platform top (38 cm wide) that was used for contact sessions (described below). When the guinea pigs were off exhibit, they inhabited a square-shaped habitat measuring 2.5 m by 1.9 m with three high walls and one low wall. The public did not have access to this habitat. While off exhibit, the guinea pigs were typically only handled to apply a quarterly, preventative topical flea medication and periodic nail trims, as needed. These treatments also occurred on exhibit. Both guinea pig habitats were near other small mammals, birds, and reptiles. All guinea pigs had multiple piles of timothy hay, water sources, feed dishes, and opaque plastic shelters for use. The animals were given fresh hay and water each morning between 0800 and 0900 and were provisioned with guinea pig pellets between 0900 and 1000. They also received produce in the afternoon at 1530. On- and off-exhibit habitats were cleaned daily.

Trained docents or zoo staff monitored interactions between the guinea pigs and the public during all operating hours. Two guinea pigs at a time were placed on the contact area for these interactions. Guinea pigs in the contact area could see, hear, and smell their group mates in the main habitat during this time. Staff and volunteers who monitored the habitat encouraged the guests to touch the guinea pigs carefully with two fingers. Each pair of animals was on the contact area for 28 min and then returned to the habitat, at which time another pair of guinea pigs was selected. Guinea pigs were selected in a set rotation such that all individuals had approximately equal exposure to public contact. Records indicated that the male guinea pigs who were on exhibit in the first 30-day period of the study participated in an average of 3.5 contact sessions per day, but, in all subsequent periods, both males and females participated in 1.5–1.9 contact sessions per day on average. This discrepancy was due to longer operating hours during the first period of our study and earlier closures of the Children’s Zoo during the fall and winter periods. Thus, guinea pigs were picked up and moved (to either the contact area in the public exhibit, or to a separate enclosure to collect fecal samples when off exhibit—see below) approximately an equal number of times in every period, with the exception of male guinea pigs during the first on-exhibit period. Guinea pigs had additional human contact from visitors touching them as described above during on-exhibit periods. We did not record the frequency or duration of actual public contact events.

The Children’s Zoo was closed to the public for 4 days from 5 September to 8 September 2017 and again for an additional 15 days from 11 September to 25 September 2017. While closed, staff continued to move guinea pigs from the on-exhibit habitat to its associated contact area, rotating between individuals every 30 min, but no visitors were present to touch them. Zoo staff still performed regular duties, cleaning, and movement through the on-exhibit area, and procedures for collection of fecal samples otherwise continued as normal.

### 2.2. Fecal Sample Collection and Extraction

Fecal pellets from exhibit animals were collected during contact sessions with visitors. In order to obtain individually identifiable fecal pellets in the off-exhibit habitats, each guinea pig in the study was removed from the group habitat and placed individually in a large plastic tub with food and a shelter for 30 min. Fecal samples were collected twice daily (1000 to 1130 and again 1300 to 1430), six days per week on exhibit and five days per week off exhibit, from July through December 2017. Sample collection was initially sporadic, and most analyses were limited to samples collected between August and December. However, samples collected in July were used to investigate how individuals responded during the transition period after changing locations (on and off exhibit). All guinea pigs were sampled during at least two complete on-exhibit and two complete off-exhibit periods, each spanning 30 days, except the final sampling period which was only 25 days (females off exhibit, males on exhibit).

Four fecal pellets (approximately 0.5 g) were required for steroid extraction and assay. Because four fecal pellets were not collected from each guinea pig every day, samples were pooled across five-day blocks. Days within blocks and sampling times (a.m. and p.m.) were evenly sampled so that the representative sample was composed of four fecal pellets from as many days and sampling times as possible. Thus, there was one pooled sample representative of each five-day block per guinea pig. Days comprising 5-day blocks did not span different sampling periods (on versus off exhibit).

All fecal pellets were frozen within 2 h of defecation and stored at −20 °C until analysis. Fecal steroids were extracted according to previously published methods [28]. Briefly, 0.5 g of fecal material was shaken overnight in 5 mL modified phosphate-saline buffer containing 50% methanol, 0.1% bovine serum albumin, and 0.5% Tween 20 (polyoxyethylene sorbitan monolaurate, a surfactant). Following centrifugation at 4000 *g* for 60 min, supernatants were decanted and stored in evaporation-proof vials at −80 °C until assay. Solid matter remaining in the extraction vials was weighed after drying overnight at 80 °C.

### 2.3. Radioimmunoassay Procedures

Fecal glucocorticoid concentrations were determined using a commercially available corticosterone radioimmunoassay (DA I-125 Corticosterone RIA, ICN MP Biomedicals, Solon, OH, USA). The lower and upper detection limits of the assay were 0.26 ng/mL and 20 ng/mL, respectively. Assays were performed according to manufacturer’s protocols with the exception that standard diluent was added to the fecal extracts, and fecal extraction buffer (containing 50% methanol) was added to the kit standards.

For all assays, standards, samples and quality control pools were assayed in duplicate. Hormone concentrations were determined as ng/mL, and then divided by the dry weight of the extracted feces to give the results as ng/g feces. Mean intra-assay variation of duplicate samples was 9.4%. Mean inter-assay coefficients of variation for two quality control pools was 8.1%.

Fecal extracts were tested for linearity by diluting 6 samples that contained high levels of hormone by 1/2, 1/4, 1/8 and 1/16 with extraction buffer. Serial dilutions were parallel to the standard curve (test of equal slopes, *p* > 0.10) [29]. The accuracy of the assay was assessed by adding a known amount of hormone to 6 fecal extracts that contained low values of hormone. Addition of known amounts of hormone at three dosage levels resulted in recovery of 104.9 ± 2.5% of added corticosterone.

### 2.4. Adrenocorticotropic Hormone (ACTH) Challenge

An adrenocorticotropic hormone challenge was performed on one male guinea pig housed at the Saint Louis Zoo. This male was received as a public donation in November 2017 and was incorporated into the ambassador program in late January 2018 after our study ended. The ACTH challenge was conducted in November 2018. Daily fecal samples were collected for 7 days prior to the procedure. On the day of the procedure, an injection of 20 IU of synthetic ACTH (Cortrosyn^®^ Amphaster Pharmaceuticals, Rancho Cucamonga, CA, USA) was administered intramuscularly. Following the procedure, fecal pellets were collected and pooled at hourly intervals for 48 h. Samples were analyzed for glucocorticoids as described above.

**Figure 1:**
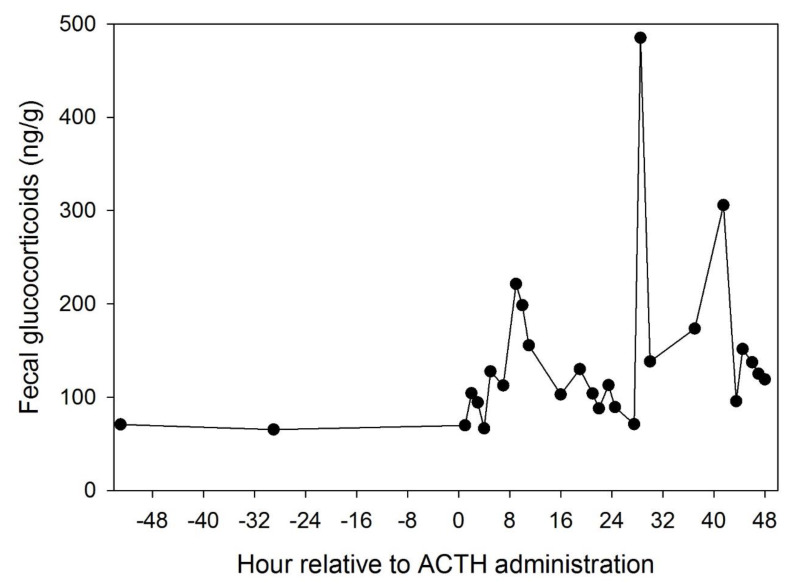
Fecal glucocorticoid profile of a male guinea pig before and after administration of adrenocorticotropic hormone (ACTH; Day 0). The maximum fecal glucocorticoid concentration was detected 28.5 h after intramuscular administration of 20 IU synthetic ACTH; concentrations returned to pre-injection levels after 43 h.

Fecal glucocorticoid concentrations prior to ACTH administration averaged 76.7 ± 4.6 ng/g (SE) (range = 58.9–91.7 ng/g). Following injection of ACTH, concentrations increased more than 6-fold, with a peak concentration of 485.0 ng/g 28.5 h after injection. Concentrations returned to baseline approximately 43 h after injection (Figure 1).

### 2.5. Weights

All guinea pigs were handled monthly to obtain a weight on a digital scale. All group members were weighed the same day. Weights are typically collected before the morning feeding and thus should not be affected by recent feeding, but the time of weight collection was not noted in the animal records. July weights were taken two days after the guinea pigs exchanged habitats, so the July weight values were assigned to the previous habitat location (on or off exhibit). September weights for the males were taken two days after an exchange so these were assigned to the previous habitat location. October weights for both sexes were taken six days after a habitat exchange so these were not included in the analyses. All other weights were taken three weeks or more after a habitat exchange and were assigned to the current habitat location at the time of weighing.

### 2.6. Data Analyses

All analyses were performed in SAS^®^ Studio 3.7 (SAS Institute Inc., Cary, NC, USA). An α = 0.05 was used to determine significant differences. The effects of sex, location, birth origin (zoo or non-zoo born), and time (habituation based on changes across sampling blocks 1 through 6 within periods) on glucocorticoid metabolite concentrations were evaluated as a nested block design using a generalized linear mixed model within the MIXED procedure with sex, location, birth origin, and the sex–location interaction as fixed factors, time as a quantitative variable nested within sex–location, and individual guinea pigs as random blocks, with heterogeneous variances within sex–location combinations. The coefficient of variation (CV) in glucocorticoid metabolite concentrations was analyzed using a similar model that excluded the effect of time, as there was a single CV calculated within each sampling period. Mean glucocorticoid levels as well as CV were analyzed as these measures may characterize different aspects of the stress response [30].

To investigate if there was an effect of moving the guinea pigs from one location to the other (on to off exhibit transition or vice versa) on glucocorticoid metabolite concentrations, a subset of the data was used that only included the values from the 5-day sampling block prior to the switch (before) and the 5-day sampling block after the switch (after). The data were evaluated as a block design using a generalized linear mixed model within the MIXED procedure with sex, time (before/after), type of switch (on to off vs. off to on), all possible interaction terms between sex, time, and type of switch, as well as birth origin as fixed factors, and individual guinea pigs as random blocks, with heterogeneous variances within sex–time combinations. A set of preplanned contrasts was used to compare a subset of the simple effect means.

CZ closures were from 5 September to 9 September and 11 September to 25 September when the females were on exhibit, so 5-day blocks considered representative of closed (vs. open) were 5 September to 9 September, 10 September to 15 September, 16 September to 20 September, and 21 September to 25 September. To see if the differences observed between the open and closed periods affected glucocorticoid metabolite concentrations in the guinea pigs present both on and off exhibit, a subset of the glucocorticoid data (all 5-day sampling blocks when guinea pigs were in the public space only) was evaluated as a nested block design using a generalized linear mixed model within the MIXED procedure with sex, CZ status (open vs. closed; 4 × 5-day sampling blocks and 19 × 5-day sampling blocks, respectively), the sex–CZ status interaction, and birth origin as fixed factors, time as a quantitative variable nested within sex, and individual guinea pigs as random blocks, with heterogeneous variances within sex. Tukey’s multiple mean comparison test was used to compare simple effect means. The off-exhibit males were also analyzed during the closures in case lack of visitor activity in the on-exhibit area somehow could be detected by the males via less noise or differences in staff activity.

Because handling rates for the males differed between their first on exhibit period compared to their other on exhibit periods, we re-ran the generalized linear mixed model for the effects of sex, location, birth origin, and time on glucocorticoid metabolite concentrations as described above, using the complete dataset (included the data from Period 1 that was previously excluded). We compared glucocorticoid metabolite concentrations between the males during their first on exhibit period to their second on exhibit period, using a linear contrast, with a one-sided null hypothesis, assuming that glucocorticoids would be higher during the first period when they were handled more frequently.

In order to compare animal weights between males and females, as well as on vs. off exhibit, weight data were analyzed using a generalized linear mixed model within the MIXED procedure with sex, location, the sex–location interaction, and birth origin as fixed factors, month and individual guinea pigs as random blocks, with heterogeneous variances within sexes. This analysis also included the first on-exhibit period from the males when handling rates were higher.

## 3. Results

### 3.1. Variation in Glucocorticoid Concentrations in Relation to Sex and Housing Location

**Figure 2:**
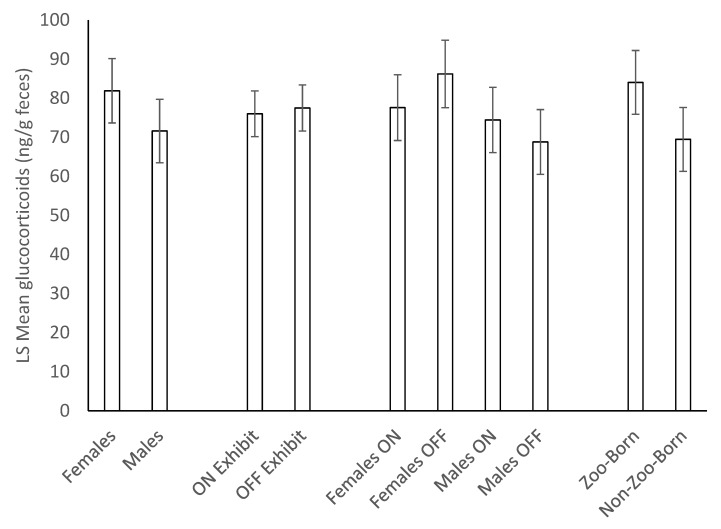
Mean ± SEM fecal glucocorticoid concentrations (N_gpigs_ = 16, N_samples_ = 344) in guinea pigs in relation to sex, birth origin, and housing location. There are no significant effects of sex, location, birth origin, or an interaction between sex and location.

**Figure 3:**
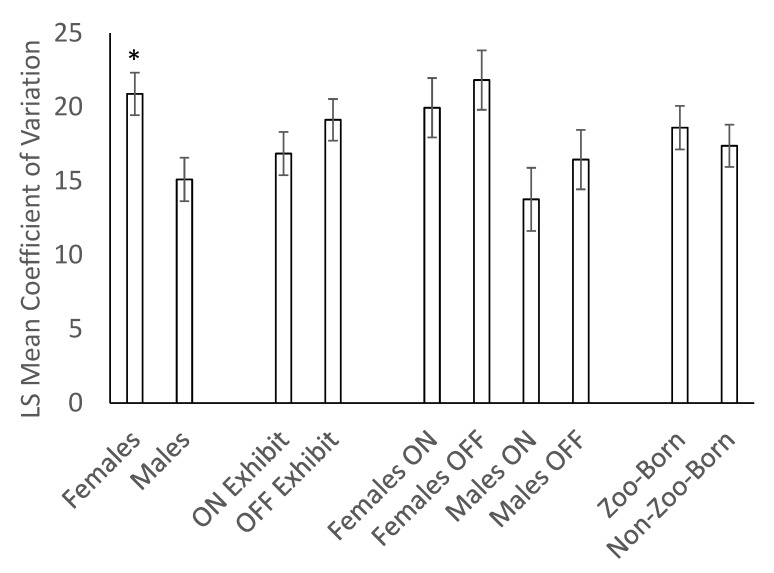
Mean coefficient of variation (CV) ± SEM of fecal glucocorticoid concentrations (N_gpigs_ = 16, N_CVs_ = 62) in guinea pigs in relation to sex, birth location, and housing location. Females displayed significantly greater variation in fecal glucocorticoid concentrations than males (*p < 0.01); no other effects were significant.

We found no effect of sex (F_1,13_ = 2.01, *p* = 0.1798), location (F_1,46_ = 0.30, *p* = 0.5895) nor a significant interaction of these variables (F_1,46_ = 0.09, *p* = 0.7674) on mean fecal glucocorticoid concentrations (N_gpigs_ = 16, N_samples_ = 344, Figure 2). Similarly, we found no effect of birth origin on mean fecal glucocorticoid concentrations (F_1,13_ = 1.54, *p* = 0.2362). However, there were significant differences in hormone concentrations among individual guinea pigs (Z = 2.38, *p* = 0.0088). We also analyzed the coefficient of variation in glucocorticoid levels to look for sex, location, and birth origin effects and found that females had significantly higher variation in glucocorticoid concentrations than males (F_1,13_ = 7.74, *p* = 0.0156, N_gpigs_ = 16, N_CVs_ = 62, Figure 3). There was no effect of location (F_1,43_ = 1.26, *p* = 0.2671), birth origin (F_1,13_ = 0.35, *p* = 0.5654), or an interaction between sex and location (F_1,43_ = 0.04, *p* = 0.8413).

**Figure 4:**
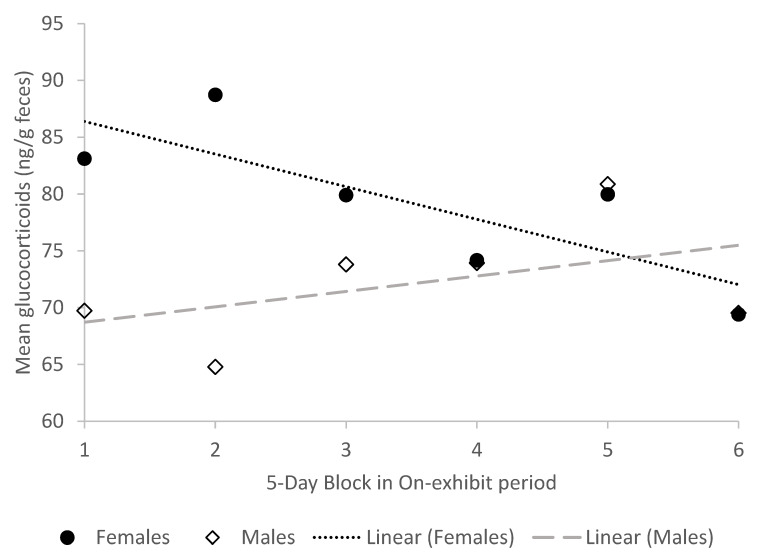
Mean fecal glucocorticoid concentrations (N_gpigs_ = 16, N_samples_ = 344) in male and female guinea pigs across five-day blocks during on-exhibit periods. There is a significant decrease in glucocorticoids in females (*p* < 0.05) and a significant increase in glucocorticoids in males over time while on exhibit (*p* < 0.05).

We investigated changes in glucocorticoid concentrations that might suggest habituation to the change in housing. We found weak but significant effects of being on-exhibit on glucocorticoid concentrations in both sexes, but the direction of the effects differed (N_gpigs_ = 16, N_samples_ = 344, Figure 4). Hormone levels decreased over time while on exhibit for females (b_1_ = −2.759, t_276_ = −2.57, *p* = 0.0106) and increased for males (b_1_ = 1.8977, t_276_ = 2.07, *p* = 0.0390). There were no significant changes in glucocorticoid levels in either sex while they were off exhibit (b_1_ = −0.5644, t_276_ = −0.33, *p* = 0.7381 and b_1_ = −0.9455, t_276_ = −1.38, *p* = 0.1691, for females and males respectively).

**Figure 5:**
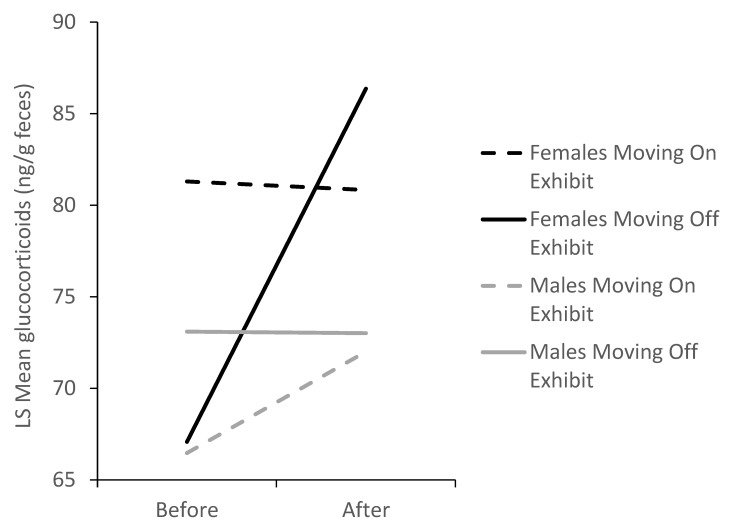
Changes in mean fecal glucocorticoid concentrations (N_gpigs_ = 16, N_samples_ = 117) in male and female guinea pigs before and after moves between on-exhibit and off-exhibit habitats. Female glucocorticoids increased significantly the week after moving to the off-exhibit habitat (*p* < 0.05); there were no other significant differences.

We compared glucocorticoid concentrations in the last block before moving the guinea pigs to the first block following the move. For each sex, we had two on-exhibit to off-exhibit transitions and two off-exhibit to on-exhibit transitions in the dataset. The effect of movement depended on the type of move and the sex of the guinea pigs (F_1,64.5_ = 3.57, *p* = 0.0632). The only significant effect detected was an increase in glucocorticoids when females were moved from on exhibit to off exhibit (t_43.5_ = 2.31, *p* = 0.0256, N_gpigs_ = 16, N_samples_ = 117, Figure 5).

### 3.2. Temporary Closure of the Children’s Zoo

**Figure 6:**
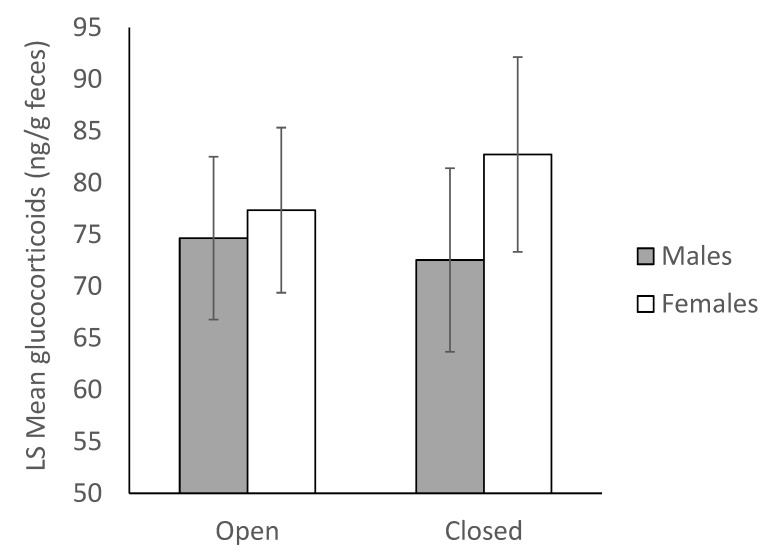
Least Squares Mean ± SEM fecal glucocorticoid concentrations during and outside of a temporary closure of the Children’s Zoo during the study (N_gpigs_ = 16, N_samples_ = 160). There was no significant effect of the closure on hormone levels in the on-exhibit females or the off-exhibit males.

We found no significant difference in glucocorticoid concentrations among male (t_124_ = −0.43, *p* = 0.9731) or female (t_124_ = 0.88, *p* = 0.8138) guinea pigs between the periods when the Children’s Zoo was open and closed (N_gpigs_ = 16, N_samples_ = 160, Figure 6).

### 3.3. Impact of Handling Rates on Glucocorticoids in Males

In order to determine whether increased handling in the first period was associated with elevated glucocorticoids, we compared the males’ glucocorticoid levels during their first and second on-exhibit periods and found no significant difference (t_55_ = 0.90, *p* = 0.1853).

### 3.4. Body Weights

There was no significant difference in body weight between males and females (males = 1334.3 g and female 1264.5 g respectively, F_1,13_ = 1.02, *p* = 0.3304), however, body weights were significantly lower when on exhibit (1286.9 g) compared to off exhibit (1311.9 g, F_1,57_ = 21.20, *p* < 0.0001). This difference represented a 1.9% difference in body weight between locations. There was no significant difference in body weight between zoo-born and non-zoo born animals (zoo-born = 1294.0 g and non-zoo born 1304.8 g respectively, F_1,13_ = 0.02, *p* = 0.8775).

## 4. Discussion

In this study, we validated a fecal assay for glucocorticoids in guinea pigs. Our result demonstrates that the fecal glucocorticoid assay used detects the appropriate metabolites for guinea pigs. The male guinea pig used for our ACTH challenge was able to mount an appropriate physiological response to exogenous ACTH treatment despite serving as an ambassador animal. This suggests that he did not have compromised adrenal function. Overall, we did not find consistent differences in glucocorticoid levels between on- and off-exhibit periods. There appear to be sex differences in glucocorticoid response to being on or off exhibit and to transitions between these habitats. Different rates of handling in males were not associated with hormonal changes. The closure of the Children’s Zoo did not have any impact on glucocorticoids for either sex, regardless of whether they were on or off exhibit during the closures. Both sexes were lighter in body weight when on exhibit, but the difference was small. On- and off-exhibit habitats differed in public exposure, physical contact and habitat size, so these factors are confounded in our study. While we cannot assign influence of the various factors on glucocorticoid levels, we can conclude that the differing experiences of these animals in on- and off-exhibit habitats do not result in differences in this possible indicator of welfare.

The most significant driver of differences in glucocorticoids observed was variation among individuals. Individual differences in physiological responses to stimuli may be due to a variety of factors, including genetics [31], age and sex [32], rearing [33], and previous experience [21]. Our data suggest male and female guinea pigs may perceive the on-exhibit habitat differently, resulting in different patterns of glucocorticoid production over time. Similarly, male guinea pigs did not appear to respond to transitions between the two habitats, whereas females responded to the transition from the on-exhibit to off-exhibit habitat with increased glucocorticoid production. Females also experienced greater glucocorticoid variability, possibly as a result of changes in glucocorticoid production during the estrous cycle [34]. While these differences could be due to sex, this factor in our study was confounded with reproductive status as all females were intact and all males were castrated. Because we had to pool feces in five-day blocks, we could not adequately assay reproductive hormones to characterize hormonal fluctuation during the estrous cycle. Regardless, our glucocorticoid results support many other studies [30,31,35,36] that emphasize the importance of individual effects on responses to the environment and thus whether certain stimuli or husbandry practices have any effect, positive or negative, on welfare.

It is not clear why the guinea pigs lost a small percentage (~2%) of their body weight while on exhibit. The larger, on-exhibit habitat could have provided more opportunity for exercise. Studies of rats and mice have demonstrated declines in body weight in response to stress [37,38,39] previous experiments on the impact of induced stress on body weights in guinea pigs have found mixed results. For example, during forced confrontations with unfamiliar males and in the presence of an unfamiliar female, male guinea pigs that had been reared with a single female lost weight over the course of the confrontations, whereas males reared in larger mixed sex colonies did not [25,26]. Female guinea pigs exposed to a startling stimulus repeatedly during pregnancy did not gain as much weight as control females during the pregnancy and weighed less during lactation [27]. Whether a loss in body weight in the absence of a rise in glucocorticoids could occur while on exhibit is unclear. Stemkens-Sevens et al. (2009) found that guinea pigs in their study lost 7%–8% of their body weight during two to eight-hour shipments and some individuals required up to 17 days to re-gain lost weight [23]. Animal care staff typically weigh the animals in the morning before providing the guinea pigs with grain, but hay and grain from the previous day are typically still available the next morning. Produce is only fed in the afternoon and is typically rapidly consumed. Animal records did not indicate the time of weighing so we cannot ascertain whether feeding times may have affected weights but our husbandry routines suggest they are weighed at least 14 hours after provision of fresh food. Review of body weights from the same period in the following year, 2018, did not reveal a significant difference between on- and off-exhibit periods (unpublished data). The small but significant difference in body weights observed in this study may be within the range of normal biological variability.

The animals in this study had been living in this management scenario for some time prior to starting the research, with the exception of the two-month old male born in the colony at the zoo. Thus, all animals had been exposed to both habitats and their associated stimuli (including visitors and contact) at least once, if not many times. All animals had also experienced at least one habitat rotation. Thus, it is possible that the overall lack of consistent responses seen is a result of habituation. Additionally, when in the contact area, the guinea pigs were with a social partner and very close spatially to their home habitat and could see, smell, and hear their other groupmates the entire time. Guinea pigs are extremely social and develop stable, peaceful relationships with group mates that provide security [12]. The fact that they were in a familiar environment with familiar partners may have reduced the likelihood of perceiving the contact area as stressful. Another possible reason for the lack of response is that guinea pigs are a domesticated species that has been bred specifically for food and pets. Previous studies of guinea pigs and their wild progenitor, the wild cavy, have found that domesticated guinea pigs have lower basal and reactive adrenal activity compared to wild cavies and show lower glucocorticoid responses to being handled for blood sampling and subsequently placed in an unfamiliar enclosure [12,40,41]. Epinephrine and norepinephrine responses to these procedures were also lower in guinea pigs compared to wild cavies. This research group also demonstrated that guinea pigs demonstrate significantly less aggressive, attentive and exploratory behavior than wild cavies and demonstrate significantly more socio-positive behavior [40,41]. Thus, the process of domestication resulted in a species that lives in calmer, more cohesive social groups and demonstrates reduced physiological responses in hypothalamic-pituitary-adrenal system and the sympathetic adrenomedullary system to stressful events. Reduction in behavioral and physiological responsiveness to potentially stressful stimuli is a common outcome of domestication [42,43,44].

While ambassador animals of exotic species could be more stimulating or engaging to guests, guinea pigs, and likely other domesticated species, offer the opportunity for safe, tactile contact that can also be engaging for children. As described above, the domestication process of multiple species resulted in animals that are adapted to, and cope better with, increased human contact, making them good candidates to serve as animal ambassadors. Indeed, many zoos already incorporate domestic animals into their ambassador programs. Previous work has demonstrated increases in glucocorticoids with increased human contact in some taxa [1], while other studies have not [4]. It is not yet clear whether the kinds of animal contact opportunities offered in professionally managed and accredited zoological facilities impact animal welfare positively or negatively (see Sherwen and Hosey, 2019, for a review, [24]). It seems most likely that these impacts will be individual specific and contingent on many different characteristics of the species selected and program operation.

While we did not find compelling evidence that guinea pigs experience negative changes in two physiological indicators of welfare, glucocorticoid production and body weight, when managed in the rotational system described, we caution that our study focused only on physiological indices and more on chronic effects rather than acute effects due to the necessity of pooling fecal samples. Future studies should focus on other indicators of welfare including behavior, physiological characteristics (e.g., heart rate variability), and other physical indicators such as coat condition. Assessments of “stress” should also include measures beyond glucocorticoids [45]. Some combination of behavioral and physiological assessments that could capture acute responses would also be instructive. Formal assessments of animal welfare were instituted in 2018 by the Association of Zoo and Aquariums, an accrediting body for zoos and aquariums. The Saint Louis Zoo assessment tool is based on the Five Domains model of welfare [46]. These assessments can be paired with future studies to ensure guinea pigs and other animals at accredited zoos and aquariums continue to experience good welfare.
